# Cell shape characteristics of human skeletal muscle cells as a predictor of myogenic competency: A new paradigm towards precision cell therapy

**DOI:** 10.1177/20417314221139794

**Published:** 2023-03-16

**Authors:** Charlotte Desprez, Davide Danovi, Charles H Knowles, Richard M Day

**Affiliations:** 1 Centre for Precision Healthcare, UCL Division of Medicine, University College London, London, UK; 2 Department of Digestive Physiology, Rouen University Hospital, Rouen, France; 3 On behalf of the EC Horizon 2020 AMELIE consortium. Details of the AMELIE consortium is provided in the Acknowledgements; 4 Centre for Gene Therapy and Regenerative Medicine, King’s College London, London, UK; 5 bit.bio, The Dorithy Hodgkin Building, Babraham Research Campus, Cambridge; 6 Blizard Institute, Centre for Neuroscience, Surgery & Trauma, Queen Mary University of London, London, UK

**Keywords:** Cell therapy, fusion index, imaging pipeline, potency, skeletal muscle

## Abstract

Skeletal muscle-derived cells (SMDC) hold tremendous potential for replenishing dysfunctional muscle lost due to disease or trauma. Current therapeutic usage of SMDC relies on harvesting autologous cells from muscle biopsies that are subsequently expanded in vitro before re-implantation into the patient. Heterogeneity can arise from multiple factors including quality of the starting biopsy, age and comorbidity affecting the processed SMDC. Quality attributes intended for clinical use often focus on minimum levels of myogenic cell marker expression. Such approaches do not evaluate the likelihood of SMDC to differentiate and form myofibres when implanted in vivo, which ultimately determines the likelihood of muscle regeneration. Predicting the therapeutic potency of SMDC in vitro prior to implantation is key to developing successful therapeutics in regenerative medicine and reducing implementation costs. Here, we report on the development of a novel SMDC profiling tool to examine populations of cells in vitro derived from different donors. We developed an image-based pipeline to quantify morphological features and extracted cell shape descriptors. We investigated whether these could predict heterogeneity in the formation of myotubes and correlate with the myogenic fusion index. Several of the early cell shape characteristics were found to negatively correlate with the fusion index. These included total area occupied by cells, area shape, bounding box area, compactness, equivalent diameter, minimum ferret diameter, minor axis length and perimeter of SMDC at 24 h after initiating culture. The information extracted with our approach indicates live cell imaging can detect a range of cell phenotypes based on cell-shape alone and preserving cell integrity could be used to predict propensity to form myotubes in vitro and functional tissue in vivo.

## Introduction

Cell-based therapy using autologous skeletal muscle derived cells (SMDC) has been technically feasible in principle for several decades. Clinical investigations conducted to date have attempted to treat a variety of conditions including muscular dystrophy, cardiac heart failure, and incontinence.^1–6^

Faecal incontinence (FI) is a condition that would particularly benefit from cell-based regenerative medicine to restore functional muscle. FI affects a large portion of the general population, with an estimated prevalence of (2%–15%).^7^ Sphincter muscle lesions are the most frequent pathophysiological alteration found in patients with this condition.^8^ Although sacral neuromodulation is currently the first-line therapy recommended after failure of conservative treatment,^9^ long-term efficacy at 10-years is achieved in less than half of patients.^10^ More definitive surgical repair of the sphincter muscle has also shown disappointing results at long-term follow-up.^11^ In terms of potential benefit, SMDC-based treatment of FI could deliver tremendous impact to the quality of life of a significant proportion of the general population affected by this common disorder.

Autologous SMDC-based therapy for FI, consisting of a mixed population of cells (but predominantly myoblasts) isolated from a biopsy taken from skeletal muscle, is reported to be safe, with 1-year efficacy in up to 80% of patients in open label studies^3,5^ and in 60% of patients in the only randomised-controlled placebo trial available to date.^6^ While these results are encouraging, the level of efficacy reported in previous studies suggests that heterogeneity in the proposed therapy may contribute to inconsistent clinical outcomes.

Improved methods for patient stratification to identify patients who are (un)likely to respond well to regenerative therapies, are needed to ensure the potential benefits of cell-based regenerative medicine can be fully exploited. To date, myogenic quality attributes of SMDC used in clinical studies for the treatment of FI have relied mainly on the use of flow cytometry analysis of the expression of the myogenic cell marker CD56 expressed throughout satellite cells and their descendants^3,6,12,13^ and muscle stem cell markers Pax7 and Myf5.^14,15^ Cell marker expression has been complemented with the use of ex vivo myotube formation assays to demonstrate competency necessary for differentiation and myofibre formation.^15,16^ More recently, the potency of SMDC has been correlated with the expression of CD56 and acetylcholinesterase activity (AChE),^16^ with AChE activity found to reflect in vitro differentiation of SMDC and the clinical potency of cells used for the treatment of FI. While these assays provide useful metrics for the batch release of SMDC products for clinical use, they involve lengthy protocols and result in the analysed cells being unusable for clinical delivery. Such approaches are not compatible with continuous monitoring of cell phenotypes in real-time, especially when limited quantities of autologous cells are available. None of the clinical or pre-clinical investigations to date exploring SMDC therapies for FI have directly evaluated the phenotypic attributes of the cells being transplanted on an individual basis using non-destructive methods that does not impinge the subsequent use of the analysed cells. Collecting this information during upstream bioprocessing and correlating it with clinical outcomes could provide invaluable information on target quality attributes during manufacture of the cell-based products.

The true extent to which isolated autologous SMDC reflect phenotypic characteristics of the donor tissue and subsequent functionality following implantation has yet to be fully elucidated. Human SMDC expansion in vitro is affected by many factors. Firstly, not all of the cells isolated from the biopsy may be compatible with ex vivo culture, resulting in a selective population of cells that preferentially grow on 2D tissue culture vessels. The culture conditions, especially culture medium, as well as background (patho)physiological characteristics of the donor, such as co-morbidity, age and gender of the donor are also likely to influence the composition of the isolated cell population.^17^ The impact of age on functionality was reported for engineered muscle derived from donors divided into three different age groups, with cells from young female donors achieving the fastest-growing SMDC in vitro and optimum contractile output of the engineered construct.^18^ These findings may shed light on how well transplanted cells function following in vivo implantation of SMDC and warrant further consideration, given that musculoskeletal conditions, such as FI, are more prevalent in the older population but also affect a smaller, much younger female population with obstetric anal sphincter injuries. If phenotypic features can be identified from in vitro behaviour of cells, it is conceivable that new methods to stratify patients most likely to benefit from cell-based therapy could be devised that would avoid treating patients in whom the regenerative response is unlikely to restore muscle function. Other factors related to the cell supply chain and manufacturing may also influence the potency of the therapy. For example, how the cell product is handled immediately before transplantation into the recipient may subsequently affect how well cells perform in vivo. Thawing of cells at point-of-care immediately before delivery is a method widely used in cell therapy studies. However, in vitro studies with skeletal muscle and other cell types indicate that achieving cell recovery with sufficient quality after cryopreservation ideally should take into consideration the optimisation of the rate of freezing and thawing to prevent osmotic shock during thawing; the state of cell differentiation at the time of cryopreservation; and inclusion of post-thaw recovery interval involving in vitro culture to increase the likelihood of subsequent good cell viability, attachment and differentiation.^19–22^

We have recently described novel analysis tools using dynamic imaging of cells in culture to provide phenotypic information at the sub-cellular, single-cell and population level using high-content image analysis.^23^ We hypothesise that a similar approach can be adopted for the development and quality control of SMDC being used for therapeutic applications. To our knowledge, the use of non-destructive multi-parametric imaging-based phenotypic characterisation of SMDC has not been attempted to date as means to assess and quantify the myogenic potency of SMDC derived from different donors. The primary aim of the study was thus precisely to evaluate the applicability of multi-parametric imaging-based phenotypic characterisation to distinguish the myogenic potency of SMDC obtained from different donors. The secondary aim was to determine whether the approach could be applied to detect phenotypic differences in myogenic potential of freshly thawed cells compared with cells that were established in culture for a short period of time in order to simulate different approaches used to handle cells prior to transplantation.

## Materials and methods

### Cell culture and cryopreservation

Commercially available human SMDC (Sk-1111) from 14 individual human donors were supplied by Cook MyoSite (Pennsylvania, USA). The cells were isolated from muscle biopsies (abdominal rectus or vastus lateralis) acquired from cadavers. Cells used in the experiments were derived from single donors and consisted of myoblast-like, non-differentiated primary human muscle cells characterised by the supplier via immunocytological analyses and/or flow cytometry of desmin and myosin heavy chain, with cells ⩾70% cells positive for desmin. The cells were cultured until Passage 2 in Ham’s F-10 Nutrient Mix (ThermoFisher Scientific) supplemented with 20% foetal bovine serum (FBS; Life Technologies Limited), 0.1 mg/ml streptomycin and 0.25 μg/ml amphotericin B (Merck), 1 µM dexamethasone (Merck) and 10 ng/ml human fibroblast growth factor (FGF)-basic (PeproTech) (proliferation medium). The culture medium was changed every 2–3 days. For subculture and harvesting, cells were rinsed once for 1 min with Dulbecco’s phosphate buffered saline (PBS; Merck) and incubated in trypsin-ethylenediaminetetraacetic acid (EDTA) solution (Merck) for 5 min at 37°C. Cells were washed in proliferation medium and centrifugated at 1000×*g* for 5 min. The supernatant was discarded and the cell pellet resuspended in 3 mL of proliferation medium before distributing into fresh tissue culture flasks.

For cryopreservation at passage 2, the cell pellet was resuspended in proliferation medium before dropwise addition of an equal volume of cryomedium (20% dimethyl sulphoxide, 40% FBS, 40% proliferation medium) and transferred into cryovials. The cell suspension was initially frozen to −80°C over a period of 24 h using a Mr Frosty™ freezing containing (ThermoFisher Scientific) before being transferred to the vapour phase of a liquid nitrogen cryostorage vessel.

### Comparison of SMDC growth

Cryopreserved SMDC at Passage 2 were thawed at 37°C in a water bath. The cell suspension was then plated in proliferation medium under the following conditions: (1) 5000 cells/cm^2^ per well (9.07 cm²) in 6 well tissue culture plates (Sarstedt) or (2) 13,000 cells/cm^2^ in a 75 cm^2^ tissue culture flasks. The culture medium was changed every 2–3 days and the cells incubated at 37°C, 5% CO_2_. Cells in the 6-well plate (1) were cultured until they reached 80%–90% confluence, at which point the culture medium was switched to ‘differentiation medium’ consisting of Dulbecco’s Modified Eagle Medium (DMEM)/F12 (ThermoFisher Scientific), 1% insulin-transferrin-selenium solution (ThermoFisher Scientific), 1% N2 supplement (ThermoFisher Scientific), 2 mM L-Glutamine (Merck) and 100 units penicillin and 0.1 mg streptomycin/mL (Merck). Cells in the 75 cm^2^ tissue culture flask (2) were cultured until they reached ~75% confluence and sub-cultured (Passage 3). The cells were replated at a density of 5000 cells/cm^2^ in a 6 well tissue culture plate (‘Passage 3 cells’) and cultured in proliferation medium until they reached 80%–90% confluence, followed by differentiation for 5 days in differentiation medium. In summary, ‘Passage 2 cells’ were considered as ‘freshly thawed cells’ and ‘Passage 3 cells’ were considered as ‘sub-cultured cells’.

For each donor, cells cultured in 6 well tissue culture plates at Passage 2 (‘freshly thawed cells’) and Passage 3 (‘sub-cultured cells’) were used for imaging, myotube formation assays and fusion index, beta-galactosidase (β-Gal) and AChE activity assay studies. Cells at Passage 3 were used for confluence studies and flow cytometry analysis of cell markers.

### Time-lapse imaging of SMDC growth

Image acquisition of SMDC growth was performed in an incubator at 37°C, 5% CO_2_ using a CytoSMART Lux2 microscope (CytoSMART Technologies B.V., Netherlands) at 20X magnification. Images of the same field of view were acquired every 5 min over a period of 3 days during the growing stage for both Passage 2 and Passage 3 cells. Each 6 well tissue culture plate was placed in the same position on the Lux2 microscope to enable imaging of the central area of the well that contained at least 20 individual cells (area of 0.92 mm × 0.92 mm, 960 × 960 pixels).

### Image analysis pipeline

**Figure 1.**
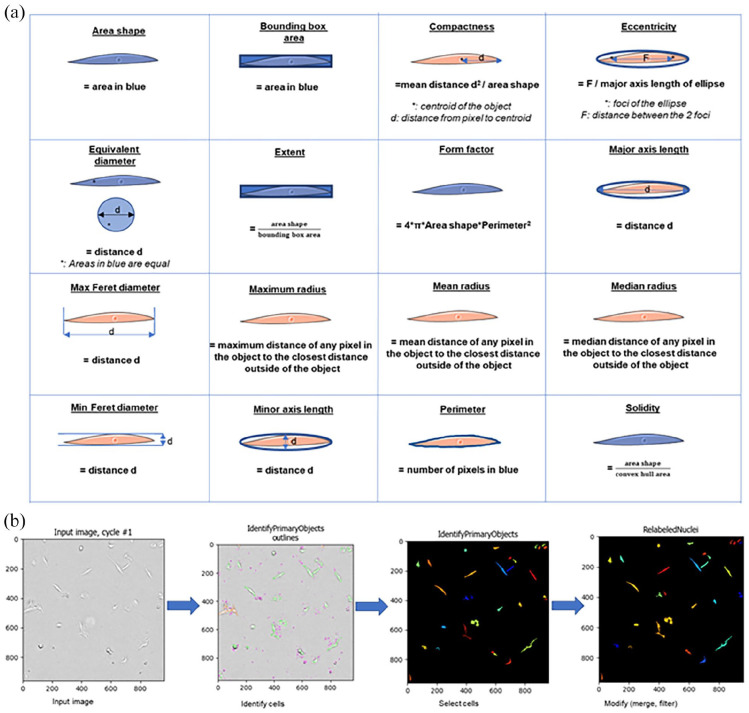
Cell shape analysis: (a) schematic representation of the different cells’ shape characteristics studies with CellProfiler software and (b) steps of the pipeline built to analyse the cells’ shape characteristics.

CellProfiler™ software (www.cellprofiler.org) version 4.1.3^24^ was used to create an image analysis pipeline that enabled the analysis of different characteristics of the cells’ shape (Table 1, Figure 1). To avoid inaccurate object identification within images, the number of objects was monitored for each image during the building of the pipeline. We first used the ‘EnhanceOrSuppressFeatures’ module with enhance neurites function to enhance the structure of the longitudinal cells and the ‘IdentifyPrimaryObjects’ module to identify the cells. For this module, the best results were obtained using 13 as minimal diameter of objects, the Otsu three classes automatic image thresholding method with pixels of the middle intensity belonging to the foreground, threshold smoothing scale of 1.3488 and without threshold correction. The lower bound of the threshold was usually 0.74 but could be adapted in case of variation of the illumination in the input image. The ‘SplitOrMergeObjects’ module was then used to merge objects within a distance of 4 pixels and the ‘FilterObjects’ module used to filter objects with major axis length under 15 pixels. Lastly, characteristics were measured using ‘MeasureObjectNeighbors’, ‘MeasureObjectSizeShape’ and ‘MeasureAreaOccupied’. The pipeline used can be found in Supporting Information Table S1. For tracking cells, a similar pipeline was built and the ‘TrackObjects’ module was used with the ‘follow neighbors’ function with an average cell diameter of 45.0 pixels and a maximal pixel distance to consider match of 70 (Supporting Information Table S1). Two hours of tracking was selected at 12 and 24 h of imaging to allow good accuracy of the pipeline to follow cells. For each characteristic studied, Konstanz Information Miner (KNIME; open-source data analytics software version 4.3.2) was used to calculate the mean and standard deviation (SD) of the characteristic for all the objects identified in one image. These data were then used for the correlation analyses.

**Table 1.** Definitions of the cell shape descriptors included in the analysis.

Characteristics	Definitions
Total Area Occupied	The number of pixels in the region occupied by all objects
Area Shape	The number of pixels in the region
Bounding Box Area	The area of a box containing the object
Compactness	The mean squared distance of the object’s pixels from the centroid divided by the area. A filled circle will have a compactness of 1, with irregular objects or objects with holes having a value greater than 1.
Eccentricity	The eccentricity of the ellipse that has the same second-moments as the region. The eccentricity is the ratio of the distance between the foci of the ellipse and its major axis length. The value is between 0 and 1. (0 and 1 are degenerate cases; an ellipse whose eccentricity is 0 is actually a circle, while an ellipse whose eccentricity is 1 is a line segment.)
Equivalent diameter	The diameter of a circle or sphere with the same area as the object.
Extent	The proportion of the pixels (2D) or voxels (3D) in the bounding box that are also in the region. Computed as the area/volume of the object divided by the area/volume of the bounding box.
FormFactor	Calculated as 4*π*Area/Perimeter2. Equals 1 for a perfectly circular object.
Major Axis Length	The length (in pixels) of the major axis of the ellipse that has the same normalised second central moments as the region.
Max Feret Diameter	The Feret diameter is the distance between two parallel lines tangent on either side of the object (imagine taking a caliper and measuring the object at various angles). The maximum Feret diameter is the largest possible diameter, rotating the calipers along all possible angles.
Maximum Radius	The maximum distance of any pixel in the object to the closest pixel outside of the object. For skinny objects, this is 1/2 of the maximum width of the object.
Mean Radius	The mean distance of any pixel in the object to the closest pixel outside of the object.
Median Radius	The median distance of any pixel in the object to the closest pixel outside of the object.
Min Feret Diameter	The Feret diameter is the distance between two parallel lines tangent on either side of the object (imagine taking a caliper and measuring the object at various angles). The minimum Feret diameter is the smallest possible diameter, rotating the calipers along all possible angles.
Minor Axis Length	The length (in pixels) of the minor axis of the ellipse that has the same normalised second central moments as the region.
Perimeter	The total number of pixels around the boundary of each region in the image.
Solidity	The proportion of the pixels in the convex hull that are also in the object, i.e. ObjectArea/ConvexHullArea.
First Closest Distance	The distance to the closest object (in units of pixels).
Second Closest Distance	The distance to the second closest object (in units of pixels).

### Confluence analysis

The confluence study was conducted using a CytoSMART Omni microscope at 10X magnification (CytoSMART Technologies B.V., Netherlands). Briefly, cryopreserved cells at Passage 3 were thawed and plated in a 6 well tissue culture plate at a density of 5000 cells/cm^2^ for 5 days in proliferation medium. The plates were imaged for 5 days and growth medium was changed every 2 days. Confluence of cells for each well was automatically calculated by the Cytosmart Omni using full plate scanning and image stitching.

### Immunostaining for myogenic markers

After 5 days of culture in differentiation medium, cells were fixed in 4% formaldehyde for 10 min. After permeabilisation (0.1% Triton-X 100 in PBS) and blocking with 5% goat serum (Merck), cells were incubated overnight with Alexa Fluor 647 conjugated anti-NCAM1 (CD56) IgG rabbit monoclonal antibody (1:500; ab237456, Abcam, United Kingdom) and MF-20 anti-sarcomeric myosin heavy chain IIa IgG mouse antibody supernatant (1:20; Developmental Studies Hydroma Bank, Iowa) in 5% goat serum. Cells were then incubated in the dark with secondary antibody (1:350 Alexa Fluor 488 IgG goat anti-mouse in 5% Goat serum). Finally, the nuclei of cells were counter stained with 4′,6-diamidino-2-phenylindole (DAPI). Immunofluorescence images were acquired with 3 channels (blue, green, red) using Leica Dmi8 microscope (Leica, United Kingdom) at 10X magnification and reconstructed using LAS X Life Science software (Leica). For each set of donor cells the navigation mode of the software was used to acquire one image at the centre of the well and four images at cardinal points from the centre of the well.

The fusion index in myotube formation assays for each donor was calculated as the ratio of nuclei number in myocytes with two or more nuclei divided by the total number of nuclei, as previously described.^25^ For each donor, five images were acquired, with one image at the centre of the well and four images at cardinal points from the centre of the well. The fusion index was calculated for each image and was generated from at least 500 randomly chosen MHC-positive cells or myotubes. The mean fusion index for each donor was calculated from the fusion index of the five images.

To analyse intracellular expression of NCAM1/CD56, a pipeline was created using CellProfiler™. The three colour channels (red, blue and green) for the acquired fluorescence microscopy images were separated using the ‘ColorToGray’ module and nuclei stained with DAPI were identified using the ‘IdentifyPrimaryObjects’ module on the blue channel. The distance between nuclei was then calculated using the ‘MeasureObjectsNeighbors’ module. The cytoplasmic intensity of NCAM1/CD56 expression was calculated for each cell using the ‘IdentifySecondaryObjects’ module and the ‘MeasureObjectIntensity’ module on the red channel. The mean NCAM1/CD56 intensity per nuclei was calculated for cultures from each donor. The correlation between the NCAM1/CD56 intensity by nucleus and the corresponding distance to the closest neighbour (=nucleus) was also calculated for each donor using Spearman correlation.

### Flow cytometry for myogenic markers

Cells cryopreserved at Passage 3 from each donor were plated in proliferation medium in 6 well tissue culture plates at a density of 5000 cells/cm^2^ and cultured until they reached 80%–90% confluence. Cells were harvested from the wells, as previously detailed, centrifugated at 1000×*g* and fixed using 4% formaldehyde. Cells were stained with FITC-conjugated anti-CD56 (Clone AF-7H3, Milteny Biotec), BV421-conjugated anti-CD34 (Clone 581, BD Biosciences) and APC-conjugated anti-CD90 (Clone 5E10, BD Biosciences) for 20 min in the dark at 4°C. Controls for non-specific staining were included consisting of isotype controls for each antibody (FITC-conjugated IS5-21F5 mouse IgG1 (Milteny Biotec, United Kingdom), BV421-conjugated anti-KLH (BD Biosciences) and APC-conjugated mouse IgG1 clone MOPC-21 (BD Biosciences). Cells were washed 2 times before the analysis. Each acquisition file included 10,000 events. A forward scatter (FSC) threshold was set to avoid debris from list mode data and for each sample. Flow cytometry analysis was conducted using BD LSRFortessa™ Cell Analyser (BD Biosciences) and BD FACSDiva™ Software for acquisition. Results were analysed using FlowJo™ version 10 software (BD Biosciences).

### SMDC senescence

Senescence of the SMDC was assessed using the senescence β-Gal staining kit (Cell Signalling Technology). Briefly, cells were cultured in proliferation medium at a density of 5000 cells/cm^2^ in a 6 well tissue culture plate for 3 days and then fixed for 15 min. The β-Gal staining solution was added to each well and the plate incubated in the dark at 37°C overnight. To ensure consistency for the experiment between all donors, cells from all donors were assayed at the same time with pH of the β-Gal staining solution controlled before use (final pH of 6.0). Assessment of positive β-Gal staining in cells was determined by the presence of blue staining colour at 24 h. Microscopy images were acquired using a ZEISS Primovert optical microscope (Zeiss) at 10X magnification with 5 fields of view per well. The number of positive cells and the total number of cells were counted manually for each image. To facilitate identification of cell staining, images were opened using ImageJ software and the Colour Threshold function applied to provide better contrast of positively stained cells. The ratio of positively stained cells divided by the total number of cells per field of view was calculated for cells from each donor.

### Acetylcholinesterase assay

Cells were plated at a density of 5000 cells/cm^2^ in 6 well tissue culture plates at Passage 2 and Passage 3 in proliferation medium until 80%–90% confluence was reached. The culture medium was switched to differentiation medium for a further 5 days culture. Analysis of AChE activity in cells from different donors was conducted using Amplite™ colorimetric AChE activity assay kit (AAT Bioquest^®^) according to manufacturer’s instructions. Briefly, 20X DTNB stock solution, 20X acetylthiocholine stock solution and AChE standard solution (50 U/mL) were prepared immediately before the experiment. The AChE working solution was prepared using 20X DNTB stock solution, 20X acetylthiocholine stock solution and assay buffer. The AChE standard solution at 50 U/mL was used to generate an AChE standard solution at 1000 mU/mL. The latter standard solution was then used to generate 7 serially diluted AChE standards (ranging from 1000 to 1.37 mU/mL) by performing 1:3 serial dilution in Assay buffer. The differentiation medium was carefully transferred from each well into a 96 well plate (50 μL per well) with one duplicate for each sample. The assay buffer solution alone was used as blank control. 50 μL of AChE working solution was added in each well of AChE standards, blank control, and test samples to make the total AChE assay volume of 100 μL/well. The reaction was incubated in the dark for 30 min at room temperature followed by the OD measurement at 405 nm on a Multiskan FC microplate absorbance reader (Thermo Scientific). For analysis, the reading obtained from the blank control well was used as negative control and this value was subtracted from the other readings to obtain the baseline corrected values. The OD measurements from the standard samples were plotted to obtain a standard curve from which AChE concentration (mU/mL) for each sample was interpolated using GraphPad Prism version 9 software.

### Statistical analyses

Mann-Whitney test for continuous variables and Chi-square test for categorical data were used for comparison among groups. Wilcoxon test was used to compare continuous paired variables. Correlation studies used the Spearman test with *r* < −0.5 or >+0.5 indicating correlation. Results are expressed as mean ± standard deviation. Data were analysed using GraphPad Prism version 9 for Windows (GraphPad Software Inc).

## Results

### Donor demographics for human SMDC

Cells isolated from 14 donors were studied and the characteristics of each donor (M/F: 5/9, mean age: 46.29 ± 22.04 years) are summarised in Table 2. No difference was seen between male and female donors regarding age (35.60 ± 12.24 vs 52.22 ± 24.56; *p* = 0.24), BMI (27.60 ± 6.34 vs 33.44 ± 7.51; *p* = 0.15), diabetes (no diabetic donors included) and tobacco use (60.00% vs 55.56%; *p* = 0.99).

**Table 2.** Characteristics of the 14 donors.

Age	Sex	Ethnicity	Cook MyoSite catalogue number	Tissue of origin	Diabetes	BMI	Tobacco use	Additional information	Fusion Index
37	M	Caucasian	01242-37M	AR	0	38	1	Non-IV drug use; Alcohol use	0.37
18	M	Caucasian	01236-18M	AR	0	21	0	Active	0.73
41	M	Caucasian	01033-41M	AR	0	25	1	Alcohol use	0.74
32	F	Caucasian	01034-32F	AR	0	42	1	NA	0.72
16	F	Caucasian	01055-16F	AR	0	37	0	Active	0.67
36	F	Caucasian	01277-36F	AR	0	26	1	Alcohol use	0.70
52	F	African American	01035-52F	AR	0	47	1	Non-IV drug use	0.69
51	M	Caucasian	01267-51M	AR	0	26	0	Non-IV drug use; Hypertension; High blood pressure; High cholesterol	0.82
31	M	Caucasian	01266-31M	AR	0	28	1	Non-IV drug use; Alcohol use	0.75
42	F	Caucasian	01269-42F	AR	0	35	1	NA	0.65
49	F	Caucasian	P01288-49F	AR	0	30	1	Pancreatitis; Non-IV drug use; Alcohol use, GERD	0.77
70	F	Caucasian	P01402-70F	VL	0	26	0	Hypertension; Osteoporosis; Asthma; Ulcerative colitis	0.49
81	F	Caucasian	P01442-81F	VL	0	32	0	Hypertension; Heart disease	0.68
92	F	Caucasian	P01520-92F	VL	0	26	0	Hypertension	0

M: male; F: female; AR: abdominal rectus; VL: vastus lateralis; NA: not applicable.

### Fusion index of SMDC correlates negatively with donor age

The fusion index calculated at day 5 of differentiation at Passage 3 is given for each cell donor in Table 2 with a mean fusion index of 0.63 ± 0.21 among all donors. SMDC from the 92-year-old donor never reached more than 50% of confluence. Therefore, the differentiation medium was not added and the fusion index was consequently considered as 0.00 for this donor. No difference was seen in terms of fusion index between male and female donors (0.69 ± 0.18 vs 0.60 ± 0.24; *p* = 0.36), suggesting gender did not influence the level of myogenicity observed. Alcohol use, tobacco use and non-IV drug use did not impact on the fusion index (with respectively *p* = 0.34, *p* = 0.28 and *p* = 0.71), indicating that lifestyle factors record for the cohort investigated were not confounding factors for myogenicity. The age of the donors negatively correlated with fusion index (*r* = −0.578; *p* = 0.03), indicating stage of life affects the propensity of the cells to form myotubes, but the BMI, a measurement of a person’s leanness or corpulence was not correlated with myogenicity (*r* = 0.111; *p* = 0.71). The total number of cells and the viability of the cells at the time of recultivation of the cells did not correlate with the fusion index (with respectively *r* = 0.384; *p* = 0.22 and *r* = 0.124; *p* = 0.70).

### Fusion index correlates negatively with CD34 expression but not with CD56 and CD90 expression

**Figure 2.**
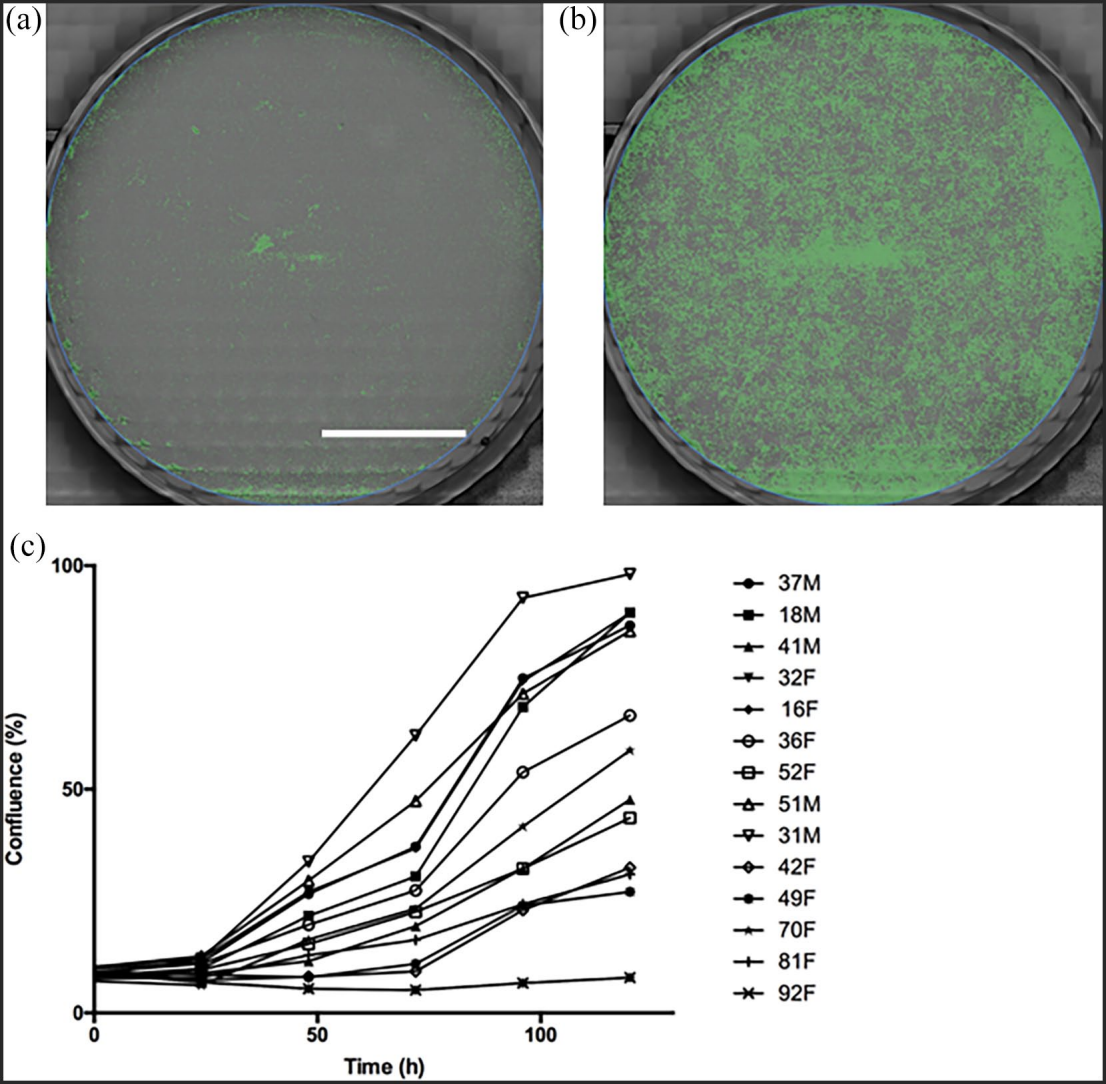
Confluence study: (a) Image of one well using the Cytosmart Omni software to automatically calculate the confluence at hour 0, (b) image of one well using the Cytosmart Omni software to automatically calculate the confluence at hour 120, and (c) graphical representation of the evolution of the confluence during time for each cell line (Bar 1 cm).

Flow cytometry revealed the mean % expression of CD56, a marker widely used for identification of human myogenic cells, across the different donors was 55.8 ± 16.76, whereas the mean % expression of CD34 was 9.98 ± 13.11 and CD90 was 94.88 ± 8.29% across the different donors (Figure 2). CD34 expression negatively correlated with the fusion index with *r* = −0.763 and *p* = 0.002, contrary to CD56 and CD90 expression (*r* = 0.131; *p* = 0.67 and *r* = −0.065; *p* = 0.83, respectively). CD34 expression did not correlate with neither CD56 expression (*r* = −0.250; *p* = 0.41) nor CD90 expression (*r* = 0.190; *p* = 0.54), but CD56 expression did correlate with CD90 expression (*r* = 0.610; *p* = 0.03). Age did not appear to be a confounding factor for myogenicity. The donor with the highest CD34 expression (43.70%) was the 92-year-old woman. However, no correlation was found between age and CD56, CD34 or CD90 expression (with respectively *p* = 0.432, *p* = 0.123 and *p* = 0.292). Lastly, the majority of CD34+ cells expressed CD56, with a mean of 0.83 ± 0.19% of CD34+CD56+ cells among CD34+ cells but no correlation was found between the percentage of CD34+CD56+ cells and fusion index (*r* = −0.192; *p* = 0.53).

### Early shape characteristics correlate negatively with the fusion index

**Figure 3.**
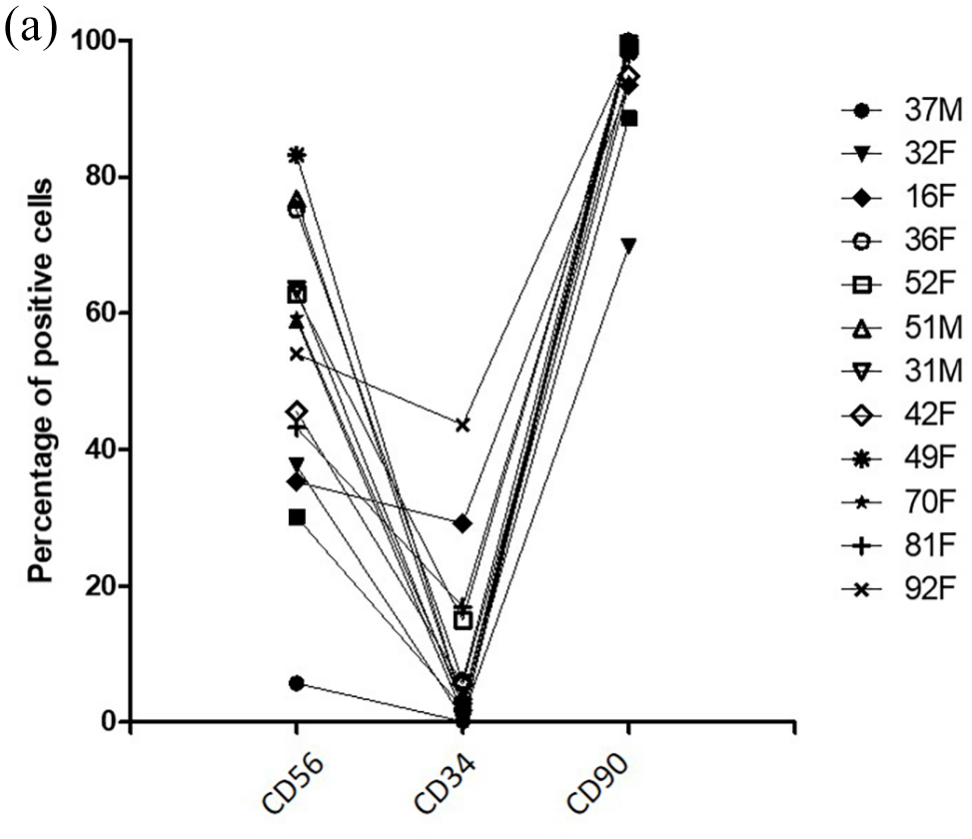
Flow cytometry analysis: (a) percentage of CD56, CD34 and CD90 positive cells for each donor.

The results for the correlation between each early shape characteristics of the cells (at 12 and 24 h of imaging during the growing phase) and the fusion index at day 5 of differentiation after Passage 3 are presented in Table 3. At 12 h of imaging, 5 cell shape characteristics were negatively correlated with the fusion index, including total area occupied by cells (*r* = −0.815, *p* = 0.001), area shape (*r* = −0.631; *p* = 0.028), bounding box area (*r* = −0.576; *p* = 0.049), minimum ferret diameter (*r* = −0.589; *p* = 0.044) and minor axis length (*r* = −0.614; *p* = 0.034), indicating the presence of cells exhibiting these shapes was associated with subsequent poorer myogenicity. Likewise, at 24 h of imaging, 8 cell shape characteristics were negatively correlated with the fusion index, including total area occupied by cells (*r* = −0.686; *p* = 0.007), area shape (*r* = −0.709; *p* = 0.005), bounding box area (*r* = −0.563; *p* = 0.036), compactness (*r* = −0.534; *p* = 0.049), equivalent diameter (*r* = −0.576; *p* = 0.031), minimum ferret diameter (*r* = −0.620; *p* = 0.018), minor axis length (*r* = −0.590; *p* = 0.026) and perimeter (*r* = −0.654; *p* = 0.011). There was a high correlation between all these characteristics at 24 h of imaging (Supporting Information Table S2). Regarding the confluence study after Passage 3 (Figure 3), no correlation was found between the confluence of the cells during the growing stage and the fusion index at 5 days of differentiation at hour 0 (*r* = 0.826, *p* = 0.068), 24 h (*r* = 0.160; *p* = 0.414), 48 h (*r* = 0.314; *p* = 0.303), 72 h (*r* = 0.252; *r* = 0.342), 96 h (*r* = 0.303; *p* = 0.310) and 120 h (*r* = 0.270; *p* = 0.330). Lastly, the distance reached by the cells using the tracking function was not correlated with the fusion index neither at 12 h (*r* = 0.070; *p* = 0.812) nor at 24 h of imaging (*r* = 0.231; *p* = 0.472).

**Table 3.** Fusion index relative to expression of CD56 after 5 days of differentiation.

Cell line	Fusion index	CD56 expression (%)
37M	0.380	5.7
18M	0.734	30.1
41M	0.743	58.9
32F	0.722	37.6
16F	0.673	35.3
36F	0.703	75.2
52F	0.694	62.8
51M	0.824	76.7
31M	0.753	63.5
42F	0.653	45.6
49F	0.767	83.2
70F	0.485	59.3
81F	0.676	43.2
92F	0.000	54.0

### Intracellular CD56 intensity per nuclei does not correlate with the fusion index but a significant correlation exists between the intracellular CD56 intensity and the distance between nuclei

**Figure 4.**
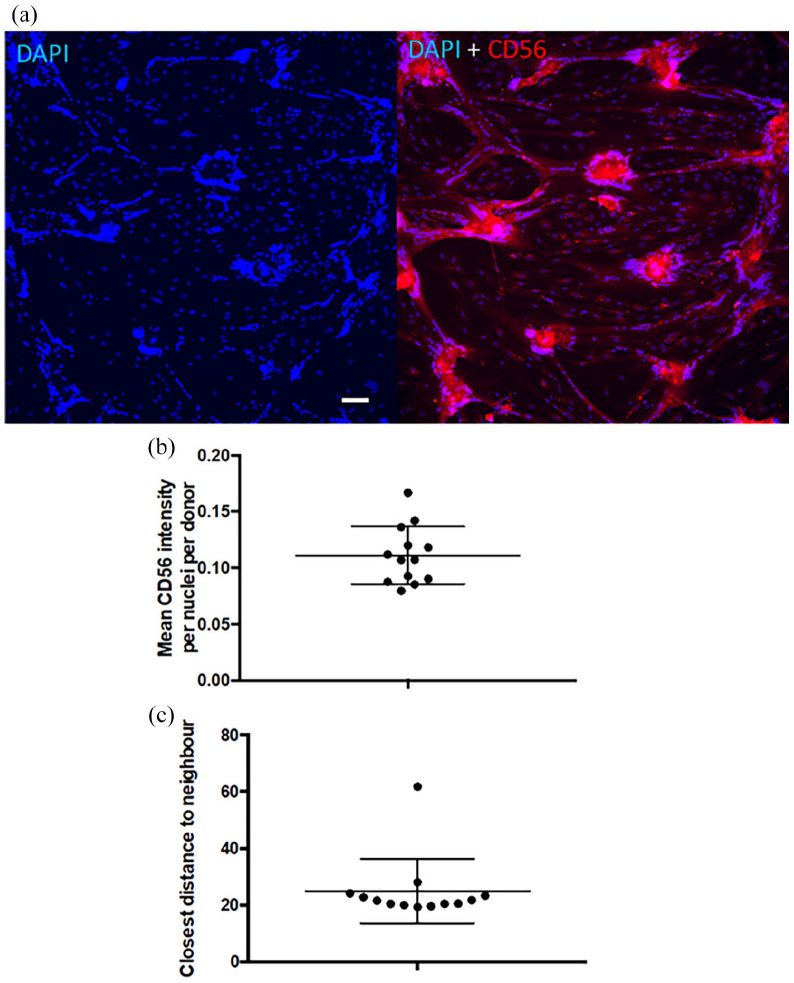
CD56 intracellular expression: (a) image taken with inverted microscope at 10X magnification with DAPI (left) and DAPI + CD56 antibody (right) validating the correlation between the distance between nuclei and the CD56 intensity (Bar 100 µm), (b) graphical representation of the mean CD56 intensity per nuclei per donor, and (c) graphical representation of the mean closest distance between nuclei for each donor.

CD56 is a reliable marker for myoblasts among cultured cells from skeletal muscle and it was reasoned that its level of expression could reflect the fusion index in cells derived from different donors. However, the mean intracellular CD56 intensity per nuclei did not correlate with the fusion index after 5 days of differentiation (*r* = 0.083; *p* = 0.786) and the range of CD56 intracellular staining intensity between donors was small (Figure 4). The closest distance between nuclei was negatively correlated to the fusion index, as expected (*r* = −0.815; *p* = 0.001). The correlation between the mean intracellular CD56 intensity and the closest distance between nuclei was calculated for cells from each donor (Table 4) and showed a statistically significant but poor correlation for all donors with the exception of the 92-year-old subject.

**Table 4.** Correlation study between early shape characteristics of the cells and the fusion index at day 5 of differentiation for Passage 2 and Passage 3.

Characteristic	Passage 2 H12		Passage 2 H24		Passage 3 H12		Passage 3 H24	
	Correlation coefficient	p	Correlation coefficient	p	Correlation coefficient	p	Correlation coefficient	p
**Total Area Occupied**	−0.285	0.345	−0.144	0.624	**−0.815**	***0.001***	**−0.686**	***0.007***
**Area Shape**	−0.553	0.050	−0.416	0.139	**−0.631**	***0.028***	**−0.709**	***0.005***
**Bounding Box Area**	−0.463	0.111	−0.202	0.488	**−0.576**	***0.049***	**−0.563**	***0.036***
**Compactness**	−0.116	0.705	−0.195	0.505	−0.441	0.151	**−0.534**	***0.049***
Eccentricity	−0.063	0.839	−0.415	0.140	0.308	0.331	−0.158	0.589
**Equivalent diameter**	−0.415	0.1259	−0.394	0.164	−0.494	0.102	**−0.576**	***0.031***
Extent	0.135	0.660	0.289	0.317	0.214	0.504	0.269	0.353
FormFactor	0.229	0.451	0.301	0.296	0.315	0.319	0.437	0.118
Major Axis Length	−0.265	0.381	−0.221	0.447	−0.375	0.230	−0.523	0.055
Max Feret Diameter	−0.243	0.424	−0.203	0.487	−0.369	0.238	−0.501	0.068
Maximum Radius	−0.545	0.054	−0.062	0.834	−0.156	0.629	−0.327	0.254
Mean Radius	−0.293	0.331	−0.022	0.940	−0.040	0.903	−0.150	0.608
Median Radius	−0.122	0.690	−0.014	0.961	−0.042	0.897	−0.048	0.871
**Min Feret Diameter**	−0.499	0.082	−0.342	0.231	**−0.589**	***0.044***	**−0.620**	***0.018***
**Minor Axis Length**	−0.441	0.132	−0.340	0.234	**−0.614**	***0.034***	**−0.590**	***0.026***
**Perimeter**	−0.307	0.308	−0.312	0.278	−0.558	0.059	**−0.654**	***0.011***
Solidity	0.300	0.319	0.212	0.467	0.309	0.329	0.404	0.152
First Closest Distance	0.406	0.169	0.282	0.329	0.257	0.420	−0.461	0.097
Second Closest Distance	0.230	0.450	−0.174	0.552	0.056	0.863	−0.160	0.584

Bold entries highlight characteristics with a significant correlation coefficient.

### β-Gal expression between donors correlates with age and CD34 expression but is negatively correlated with fusion index

**Figure 5.**
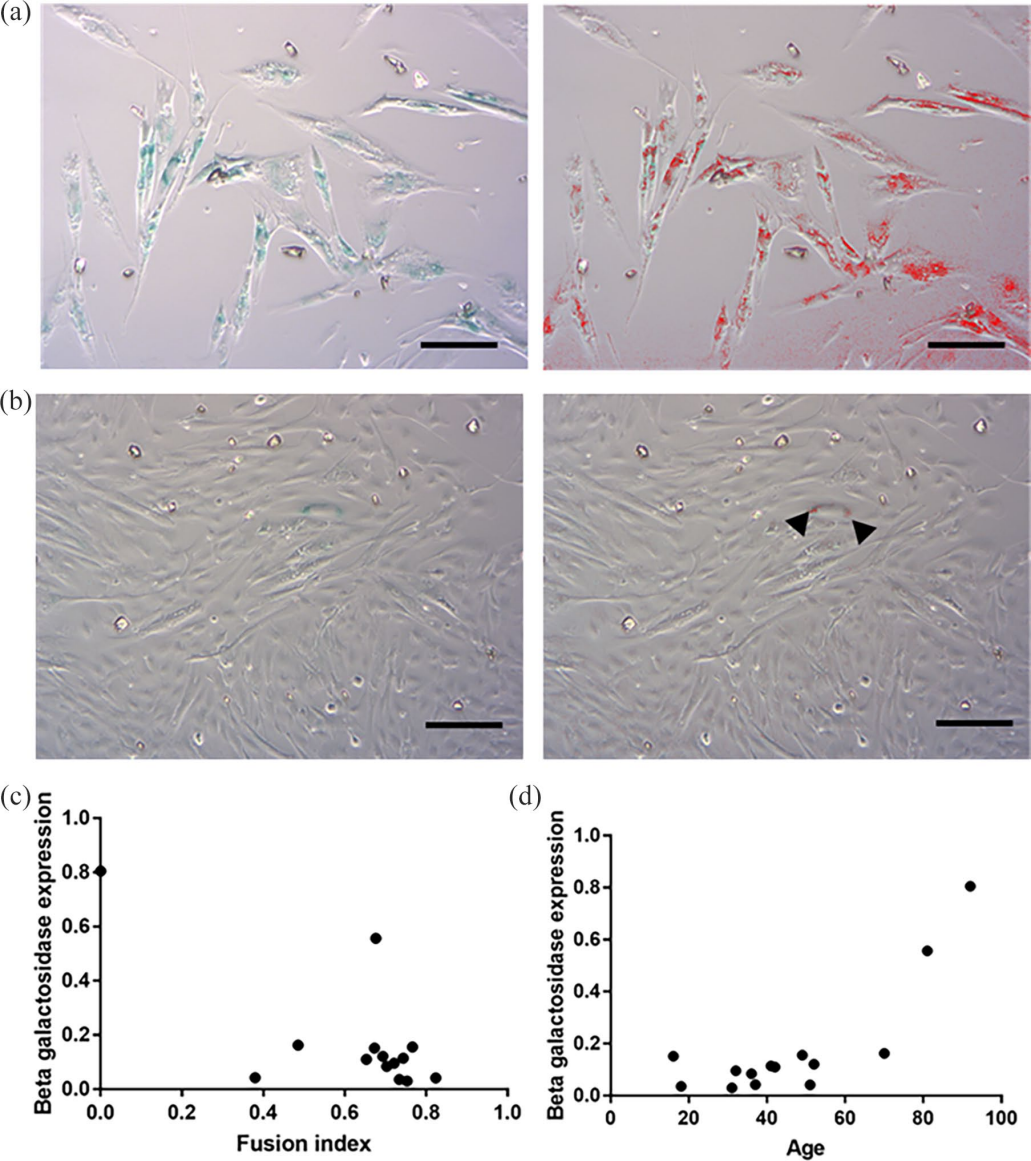
β-Gal expression. (a) Image of growing SkMDC cells from the 92-year-old donor taken with an optical microscope at 10X magnification. The strong blue colouration shows a high expression of β-Gal by 80.52% of cells (left image) and the same image after use of ImageJ software to identify the blue staining using the Colour threshold function. (b) Image of growing SkMDC cells from the 41-year-old donor taken with an optical microscope at 10X magnification. The strong blue colouration shows expression of β-Gal by 4.24% of cells (left image) and the same image used with ImageJ software to identify the blue colouration using the Colour threshold function (black arrow heads). (Bar 100 µm) (c) Graphical representation of the B-Gal expression in function of the fusion index after 5 days of differentiation for each cell line showing a significant negative correlation (*r* = −0.695; *p* = 0.006). (d) Graphical representation of the B-Gal expression in function of the age of the donor showing a significant positive correlation (*r* = −0.695; *p* = 0.006).

Detection of β-Gal expression can be used to identify senescent cells in heterogeneous cell populations. The mean percentage of cells expressing β-Gal from different donors was 17.95 ± 22.26% at Passage 3 (Figure 5). The fusion index after 5 days of differentiation was negatively correlated with the percentage of cells expressing β-Gal (Figure 5(c); *r* = −0.695; *p* = 0.006). A positive correlation was found between the percentage of cells expressing β-Gal and the age of the donor (Figure 5(d); *r* = 0.808; *p* = 0.0005). No correlation was found between BMI of donors and the percentage of cells expressing β-Gal (*r* = −0.114; *p* = 0.698). The percentage of cells expressing β-Gal was significantly lower in male donors in comparison to female donors (5.34 ± 0.03% vs 24.96 ± 25.38%; *p* = 0.007). No difference was seen when comparing the percentage of cells expressing β-Gal in donors with alcohol use and in those without (*p* = 0.282), the same for tobacco use (*p* = 0.298) or for non-IV drug use (*p* = 0.240). Lastly, a positive correlation was found between the percentage of CD34+ SMDC and the percentage of cells expressing β-Gal among donors (*r* = 0.797; *p* = 0.001), but not with the percentage of CD56+ (*r* = −0.143; *p* = 0.640) or CD90+ (*r* = 0.166; *p* = 0.588) SMDC.

### Acetylcholinesterase activity does not correlate with fusion index

AChE activity was evaluated in cells from 11 donors. The mean AChE activity after 5 days of differentiation among cell lines was 10.12 ± 5.83 mU/mL. AChE activity did not correlate with age (*r* = −0.243; *p* = 0.059) or BMI (*r* = −0.340; *p* = 0.096) and no difference was found for AChE activity with use of alcohol (*p* = 0.429), tobacco (*p* = 0.92) or non-iv drug use (*p* = 0.083). Although AChE has previously been suggested to correlate with clinical potency of SMDC, no correlation was found between AChE activity and the fusion index after 5 days of differentiation (*r* = 0.2859; *p* = 0.394). The percentage of CD56+ (*r* = 0.180; *p* = 0.596), CD34+ (*r* = 0.031; *p* = 0.927) and CD90+ (*r* = 0.495; *p* = 0.122) growing SMDC did not correlate with the AChE activity. There was a tendency of a negative correlation with the β-Gal expression with *r* = −0.572 IC_95%_ [−0.873 to 0.042] (*p* = 0.327).

### β-Gal and CD34 expression correlate with surface area and size of cells

A supplementary analysis was performed to see if any cell shape characteristics correlate with the other markers studied (β-Gal, CD56, CD34, CD90 and AChE activity) is reported in Table 5. Among all the cell shape characteristics, the area shape (*r* = 0.645; *p* = 0.012), the compactness (*r* = 0.698; *p* = 0.005), the form factor (*r* = −0.548; *p* = 0.047), the minor axis length (*r* = 0.646; *p* = 0.013) and the perimeter (*r* = 0.687; *p* = 0.007) were correlated with β-Gal expression. The same characteristics also correlated with the CD34 (Table 6), but also the major axis length (*r* = 0.624; *p* = 0.017). No characteristic correlated with the CD56 and the CD90 expression and only the form factor correlated with the AChE activity (*r* = 0.651; *p* = 0.030; Table 6).

**Table 5.** Correlation studies between the mean intracellular CD56 intensity per nuclei and closest distance between nuclei for each donor.

	*r*	*p*
37M	−0.219	<0.0001
18M	−0.276	<0.0001
41M	−0.270	<0.0001
32F	−0.252	<0.0001
16F	−0.146	<0.0001
36F	−0.294	<0.0001
52F	−0.397	<0.0001
51M	−0.241	<0.0001
31M	−0.253	<0.0001
42F	−0.247	<0.0001
49F	−0.124	<0.0001
70F	−0.242	<0.0001
81F	−0.232	<0.0001
92F	−0.138	0.09

**Table 6.** Correlation of cell shape characteristics with the results of the senescence testing, flow cytometry and AChE activity.

	Area shape		Bonding box area		Compactness		Eccentricity		Equivalent Diameter		Extent		Form Factor		Major Axis Length		Max Feret Diameter	
	*r*	*p*	*r*	*p*	*r*	*p*	*r*	*p*	*r*	*p*	*r*	*p*	*r*	*p*	*r*	p	r	p
β-Gal expression	**0.645**	0.013*	0.516	0.059	**0.698**	0.005*	0.066	0.821	0.523	0.055	−0.332	0.246	−0.538	0.047*	0.485	0.079	0.468	0.092
CD56 expression	0.131	0.671	−0.003	0.993	−0.005	0.986	−0.293	0.332	0.301	0.318	0.055	0.859	0.039	0.900	0.089	0.773	0.055	0.858
CD34 expression	**0.644**	0.013*	**0.574**	0.032*	**0.633**	0.015*	0.170	0.561	**0.564**	0.036*	−0.430	0.124	−0.558	0.038*	**0.625**	0.017*	**0.598**	0.024*
CD90 expression	−0.095	0.746	−0.192	0.511	−0.299	0.299	0.173	0.555	−0.022	0.940	0.114	0.698	0.049	0.868	−0.027	0.927	−0.088	0.765
AChE activity	0.116	0.735	−0.415	0.205	−0.352	0.288	−0.589	0.057	−0.381	0.247	−0.279	0.405	**0.651**	0.030*	0.585	0.059	−0.331	0.320
	Maximum Radius		Mean Radius		Median Radius		Min Feret Diameter		Minor Axis Length		Perimeter		Solidity		First Closest Distance		Second Closest Distance	
	*r*	*p*	*r*	*p*	*r*	*p*	*r*	*p*	*r*	*p*	*r*	*p*	*r*	*p*	*r*	p	r	p
β-Gal expression	0.151	0.607	−0.037	0.900	−0.089	0.762	**0.658**	0.011*	**0.646**	0.013*	**0.687**	0.007*	−0.600	0.023*	0.225	0.438	−0.066	0.822
CD56 expression	0.045	0.884	0.172	0.575	0.257	0.397	0.219	0.472	−0.190	0.535	0.035	0.909	−0.132	0.668	−0.011	0.972	0.030	0.922
CD34 expression	0.170	0.562	0.000	1.000	−0.019	0.948	**0.609**	0.021*	**0.578**	0.030*	**0.670**	0.009*	−0.558	0.038*	0.011	0.971	−0.190	0.515
CD90 expression	0.159	0.586	0.220	0.450	0.167	0.569	−0.108	0.712	−0.173	0.555	−0.206	0.479	0.148	0.614	−0.242	0.405	−0.283	0.326
AChE activity	−0.387	0.240	0.266	0.430	0.397	0.227	0.343	0.302	−0.323	0.332	−0.528	0.095	−0.525	0.097	0.371	0.261	0.493	0.124

* *p* < 0.05.

Shaded bold entries highlight characteristics with a significant correlation coefficient.

### Fusion index does not differ between freshly thawed cells and cells already established in culture but freshly thawed cells take longer to reach confluence

**Figure 6.**
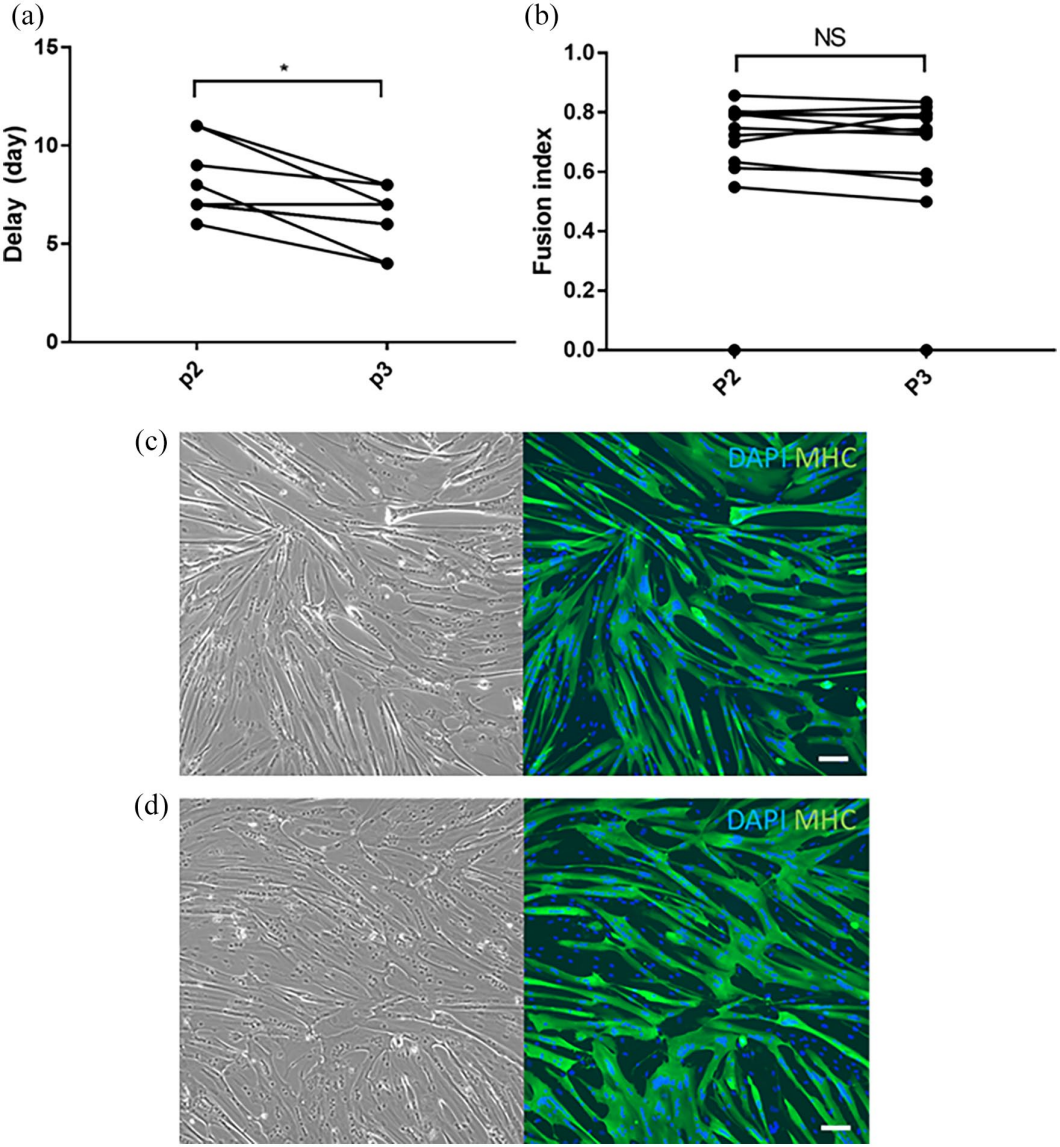
Comparison of fusion index at day 5 between defrosted cells and sub-cultured cells. (a) Comparison of the delay to reach 80% of confluence between defrosted cells (P2) and subcultured cells (Passage 3). The delay was higher in defrosted cells than in subcultured cells with *p* = 0.008. (b) Comparison of the fusion index at 5 days of differentiation between defrosted cells (Passage 2) and subcultured cells (Passage 3). No difference was observed between the 2 groups (*p* = 0.28). (c) Image taken with inverted microscope at 10X magnification and with blue and green filters to identify DAPI and myosin heavy chain intracellular expression of defrosted cells from the 32-year-old woman donor. The fusion index was 0.72. (d) Image taken with inverted microscope at 10X magnification and with blue and green filters to identify DAPI and myosin heavy chain intracellular expression of subcultured cells of the 32-year-old woman donor. The fusion index was 0.73. (Bar 100 µm).

The delay to reach ~80% confluence during the cell expansion phase (necessary before adding differentiation medium) was greater in the freshly thawed cells than in sub-cultured cells (7.91 ± 1.70days vs 6.35 ± 1.36 days; *p* = 0.008; Figure 6). SMDC from the 92-year-old donor never reached more than 50% of confluence in proliferation medium, so this donor was excluded from the analysis. The mean fusion index at day 5 of differentiation was 0.67 ± 0.23 for the freshly thawed cells and 0.66 ± 0.23 for subcultured cells (*p* = 0.28; Figure 6).

### Cell shape characteristics differ between freshly thawed cells and sub-cultured cells

As the pipeline was efficient in identifying cells that do not overlap, the number of cells identified from frame to frame was calculated during the 3 days of imaging for the freshly thawed cells (Passage 2) and sub-cultured cells (Passage 3). The time to double the initial number of objects by frame was 44.90 ± 17.42 h for sub-cultured cells versus 65.41 ± 17.40 h for freshly thawed cells (*p* < 0.001), showing that cells grow and overlap faster in sub-cultured cells. Moreover, no correlation was found between the cell shape characteristics of the freshly thawed cells at both 12 and 24 h of imaging and the fusion index at day 5 of differentiation (Table 3). Another analysis showed that almost all the cell shape characteristics differed between freshly thawed cells and sub-cultured cells at both 12 and 24 h of imaging (Supporting Information S4 Appendix), with the exception of the minor axis length, mean and maximum radius. At 24 h of imaging, the freshly thawed cells seemed to be smaller (in both length and width), more circular and less close to each other than the sub-cultured cells. Twelve of the 19 cell shape characteristics of the freshly thawed cells at 24 h of imaging were correlated with those of the sub-cultured cells at the same time of imaging (Supporting Information Table S3). Lastly, the distance reached by cells was higher in sub-cultured cells than in freshly thawed cells at both 12 and 24 h of imaging (with respectively *p* < 0.001 and *p* < 0.001; Supporting Information S4 Appendix).

AChE activity in SMDC after 5 days of differentiation did not differ between freshly thawed cells and sub-cultured cells (11.36 ± 3.19 mU/mL in the freshly thawed cell group versus 10.12 ± 1.76 mU/mL in sub-cultured cell group (*p* = 0.99)). Lastly, no difference was seen regarding the mean percentage of cells expressing β-Gal between freshly thawed cells and sub-cultured cells after 5 days of differentiation, with respectively 0.20 ± 0.06 and 0.17 ± 0.06; *p* = 0.93.

## Discussion

The inability to predict potency of autologous cell-based products before they are implanted into patients hinders development of therapies and may prevent elucidation of the underlying mechanistic effects that can be attributed to clinical outcomes observed. The use of quality attributes for cell-based products that focus solely on the use of cell marker expression rather than biomarkers that reflect the ultimate intended functional properties of the resultant tissue is likely to be a contributing factor in the heterogenous outcomes of clinical studies. The inability to stratify patients into groups that identifies those likely to respond best is costly and can lead to the abandonment of potentially life-changing new treatments. This is particularly relevant for tissue replacement therapies, where the product being delivered comprises of parenchymal cells, such as autologous SMDC in the treatment of FI, that are intended to directly replace or restore definitive function of the tissue.

In the present study, we have shown that the SMDC fusion index is negatively correlated to the age of the donor, which is in line with findings previously reported that separated patients among three age groups (20–39 years; 40–59 years and 60–80 years).^18^ This result is supported by the negative correlation between β-Gal expression and the fusion index at a cellular level, even at low passage. Indeed, the donor with the highest β-Gal expression (more than 80% of cells) was the 92-year-old subject. We found no difference in terms of fusion index between sex, which differs from previous report,^18^ where young female donors were associated with fast-growing, functional cells. It is possible that our result could be explained by a lack of power, since only 5 of the 14 donors were men in the present study. Furthermore, the donors in our study were not strictly ‘healthy’ volunteers based on the reported abnormal BMI, alcohol, non-IV drug or tobacco use, which might affect the quality of the SMDC via oxidative modifications of proteins.^26,27^ Although many of these characteristics were correlated with fusion index outcomes, the results should be interpreted with caution.

Several of the early cell shape characteristics were found to negatively correlate with the fusion index. These included total area occupied by cells, area shape, bounding box area, compactness, equivalent diameter, minimum ferret diameter, minor axis length and perimeter of SMDC at 24 h after initiating culture. The results indicate that monitoring of cell shape during the early stages of bioprocessing using real-time imaging could be used to predict cellular competency necessary for differentiation and myofibre formation in vivo, which in turn could help with selection of either patients to treat or cell populations more likely to yield better outcomes in cell-based therapy. Confirmation of the ability of cell shape characteristics in vitro to predict clinical efficacy requires further pre-clinical in vivo exploration that will enable testing of functional outcomes, such as cell engraftment and force generation in treated muscle following transplantation of cells. Monitoring of myogenicity markers in vivo alongside to cell shape characteristics would also be interesting to confirm the heterogeneity of cells population.

The methodology used in the current study is rapid and non-destructive, relying on the shape of the cells in images acquired using bright field microscopy. To our knowledge, no similar study on human cells have been performed to date, whereas one used an immortalised mouse cell line (C2C12) to predict myotube formation.^28^ Consistent with our study, results indicated the width, length and perimeter of cells being important parameters during monitoring that could distinguish between different experimental conditions. However, in the current study these characteristics were positively correlated with β-Gal expression. Senescent cells are known to increase in size, which is in line with our results.^29^

We did not find any correlation between the CD56+ membrane expression of SMDC and the fusion index. This observation also coincided with a lack of correlation between cell shape characteristics and CD56 expression. The mean level of CD56+ expression was 55.8 ± 16.76% in the present study, in line with previous reports,^30,31^ where the expression was between 50% and 80% of the total cell population. When separating myoblasts on basis of CD56 expression, CD56+ myoblasts were previously shown to better fuse into myotubes in comparison to CD56 negative cells, where only a low number of myotubes were observed.^30^ Unlike previous studies, the current study did not separate cells according to CD56 expression prior to conducting the fusion index, therefore the mixed population of cells investigated might have contributed to the lack of correlation observed. The brightfield microscopy imaging system used to acquire cell images in the current study was not compatible with cell staining to distinguish myogenic cells in the mixed population. Future studies are planned to incorporate this feature so that shape characteristics in the context of myogenic cell population can be distinguished. Furthermore, CD34 membrane expression correlated negatively with the fusion index in our study. Few studies have assessed CD34 expression in human skeletal myogenesis. One report found that the CD34 expression had no impact on the myogenic potential of human myoblasts in cell populations containing a similar percentage of CD56+ cells.^32^ In the same study, loss of CD34 expression was associated with loss of adipogenic potential of cells. Another report found that high expression of CD34 in stem cells was associated with stemness properties and that low CD34 expression was more associated with myogenic differentiation.^33^ It is possible that CD34+ cells are less engaged in myogenic differentiation than the CD34-cells and could stay in an inactivated quiescent state, explaining the inverse correlation with the fusion index.^34^ Lastly, we found no correlation between the fusion index and the AChE activity in the present study. AChE is a type B carboxylesterase found in skeletal muscle that is primarily active in cholinergic synapses and neuromuscular junctions.^35–38^ Previously, AChE activity has been reported to be low in proliferating myoblasts but increased following differentiation and fusion of SMDC.^39,40^ Our findings contrasted the prior studies, where the AChE activity was correlated to the fusion index.^16^ The reason for this is unclear and requires further investigation. The cells used in the current study and previous study by Thurner et al. are primary cultures derived from different commercial sources, which is likely to give rise to different confounding factors associated with cell donor demographics as well as differences in the way cells have been processed. A limitation of our study could be that the 92-year-old donor was excluded from this analysis as we did not manage to grow enough cells for this experiment. Secondly, the density of cells used for our experiments was lower (48,000 cells per well, 6-well plate) compared with density used in the latter study (120,000 cells per well, 24-well plate).^16^

Differences in shape characteristics were observed between freshly thawed and sub-cultured cells. We observed that the distance reached by the cells and almost all the cells shape characteristics were different between our two groups. This difference may be due to the effect of freezing the cells but did not impact the differentiation potential once growth was established, since there was no observable difference in the fusion index. Neither was there a difference in AChE activity in SMDC between freshly thawed cells and sub-cultured cells. The impact of subculturing cells beyond P3 was not investigated in the current study but the impact of higher passaging on cell shape descriptors in relation to the onset of cell senescence would be worthy of further exploration. This finding is in accordance with previous reports that compared fresh cells with frozen SMDC.^31,35^ Indeed, primary myoblast cultures can be effectively established from tissue cryopreserved for various lengths of time.^41^

## Conclusion

Results from the current study indicate cell shape descriptors applied to images non-destructively acquired during cell culture could be used as markers to predict potency of SMDC in replenishing muscle function prior to implantation. Cell-based therapies are being explored for a number of muscle disorders, including cardiac disease, incontinence, dystrophies and volumetric muscle loss associated with trauma. It is reasonable to predict that similar non-destructive imaging-based approaches could be applied to isolated cells intended to treat these conditions following identification cell-shape descriptors that correlate with established markers of potency. The method is simple and low-cost, enabling it to be incorporated into existing bioprocessing regimes. The findings present a novel approach and further studies conducted in conjunction with clinical investigations exploring SMDC-based therapies could establish whether selection of either patients or cell populations on this basis have a higher probability of yielding better outcomes in cell-based therapies.

## Supplemental Material

sj-docx-1-tej-10.1177_20417314221139794 – Supplemental material for Cell shape characteristics of human skeletal muscle cells as a predictor of myogenic competency: A new paradigm towards precision cell therapy Click here for additional data file. Supplemental material, sj-docx-1-tej-10.1177_20417314221139794 for Cell shape characteristics of human skeletal muscle cells as a predictor of myogenic competency: A new paradigm towards precision cell therapy by Charlotte Desprez, Davide Danovi, Charles H Knowles and Richard M Day in Journal of Tissue Engineering
