# Supplementary material for: Cell shape characteristics of human skeletal muscle cells as a predictor of myogenic competency: A new paradigm towards precision cell therapy
Source: J Tissue Eng. 2023 Mar 16;14:20417314221139794. doi: 10.1177/20417314221139794 (PMC10026113; doi:10.1177/20417314221139794)

Supporting Information

S1 Table. Pipeline.

>Images

>Metadata

Yes>Extract from image file headers

Extract data from all images

>NamesAndTypes

Assign a name to all images>Process as 3D>No

Select the image type>Grayscale image

>Groups

Do you want to group your images>No

>EnhanceOrSuppressFeatures

Select the operation “Enhance”

Feature type “Neurites”

Enhancement method “Line structure”

Feature size “40”

Rescale result image>No

>Identify primary objects

Typical diameter in pixels units (Min, Max) “13” “1000”

Discard objects outside the diameter range>Yes

Discard objects touching the border or the image>Yes

Threshold strategy “Global”, Thresholding method “Otsu”, “Three class”, “Foreground”, Threshold smoothing scale “1.3488”, Threshold correction factor “1.0”, lower and upper bounds on threshold “0.74-1.0”, No log transformation, No method to distinguish clumped objects

>SplitOrMergeObjects

Input objects=IdentifyPrimaryObjects, Name the new objects “relabeled nuclei”, Operation “Merge”, Merging method “Distance”, Max distance to merge objects “4”

>MeasureObjectsSizeShape

Measure “relabeled nuclei”

>FilterObjects

Filter relabeled nuclei, Method “measurements”

Select the filtering method “limits”, “area shape”, “Major axis length”

Filtering using a minimum value “yes”, minimum value “15”

Filtering using a maximum value “no”.

>MeasureObjectsNeighbors

Select objects “FilterObjects”, Neighbors “FilterObjects”

Method “Expend until adjacent”

Consider objects discarded for touching border “yes”

>MeasureObjectSizeShape

Select “FilterObjects”

>MeasureImageAreaOccupied

Select “FilterObjects”

>TrackObjects

Method “follow neighbors”, Select “FilterObjects”, average cell diameter “45.0”, maximum pixels to consider match “70”.

>ExportToSpreadSheet

S2 Table. Correlation between cell shape characteristics at 24 hours of imaging at Passage 3.

| R coefficients | **Total area** | **Area shape** | **Bonding box area** | **Compactness** | **EquivalentDiameter** | **MinFeretDiameter** | **MinorAxisLength** | **Perimeter** |
| --- | --- | --- | --- | --- | --- | --- | --- | --- |
| **Total area** |  | **0,8607202** | **0,7496722** | **0,5426071** | **0,8655726** | **0,8752095** | **0,8434718** | **0,7759587** |
| **Area shape** | **0,8607202** |  | **0,9298015** | **0,7393303** | **0,9637382** | **0,9291487** | **0,9161186** | **0,9529547** |
| **Bonding box area** | **0,7496722** | **0,9298015** |  | **0,8131936** | **0,9007918** | **0,8573657** | **0,8456472** | **0,951126** |
| **Compactness** | **0,5426071** | **0,7393303** | **0,8131936** |  | **0,6626793** | **0,7807855** | **0,7713336** | **0,906086** |
| **EquivalentDiameter** | **0,8655726** | **0,9637382** | **0,9007918** | **0,6626793** |  | **0,9306623** | **0,9176339** | **0,9048481** |
| **MinFeretDiameter** | **0,8752095** | **0,9291487** | **0,8573657** | **0,7807855** | **0,9306623** |  | **0,994315** | **0,9310178** |
| **MinorAxisLength** | **0,8434718** | **0,9161186** | **0,8456472** | **0,7713336** | **0,9176339** | **0,994315** |  | **0,9193881** |
| **Perimeter** | **0,7759587** | **0,9529547** | **0,951126** | **0,906086** | **0,9048481** | **0,9310178** | **0,9193881** |  |

| P values | **Total area** | **Area shape** | **Bonding box area** | **Compactness** | **EquivalentDiameter** | **MinFeretDiameter** | **MinorAxisLength** | **Perimeter** |
| --- | --- | --- | --- | --- | --- | --- | --- | --- |
| **Total area** |  | **7,7543E-05** | **0,00202045** | **0,04498224** | **6,3374E-05** | **4,1449E-05** | **0,00015022** | **0,00110484** |
| **Area shape** | **7,7543E-05** |  | **1,4833E-06** | **0,00251338** | **3,0353E-08** | **1,5657E-06** | **4,189E-06** | **1,4138E-07** |
| **Bonding box area** | **0,00202045** | **1,4833E-06** |  | **0,00040489** | **1,1082E-05** | **8,8767E-05** | **0,00013881** | **1,7703E-07** |
| **Compactness** | **0,04498224** | **0,00251338** | **0,00040489** |  | **0,00980264** | **0,00098053** | **0,00123546** | **8,0691E-06** |
| **EquivalentDiameter** | **6,3374E-05** | **3,0353E-08** | **1,1082E-05** | **0,00980264** |  | **1,38E-06** | **3,7676E-06** | **8,7047E-06** |
| **MinFeretDiameter** | **4,1449E-05** | **1,5657E-06** | **8,8767E-05** | **0,00098053** | **1,38E-06** |  | **4,815E-13** | **1,3392E-06** |
| **MinorAxisLength** | **0,00015022** | **4,189E-06** | **0,00013881** | **0,00123546** | **3,7676E-06** | **4,815E-13** |  | **3,324E-06** |
| **Perimeter** | **0,00110484** | **1,4138E-07** | **1,7703E-07** | **8,0691E-06** | **8,7047E-06** | **1,3392E-06** | **3,324E-06** |  |

S3 Table. Correlation between cell shape characteristics of the freshly thawed cells (P2) with sub-cultured cells (P3) at the same time of imaging.

| **Area Shape** | P2 H12 | P3 H12 | P2 H24 | P3 H24 |  | Coeff | P2 H12 | P3 H12 | P2 H24 | P3 H24 |
| --- | --- | --- | --- | --- | --- | --- | --- | --- | --- | --- |
| P2 H12 |  | 0.02035821 | 0.6529903 | 0.1054832 |  |  |  | 0.6324742 | 0.1380058 | -0.4695366 |
| P3 H12 | 0.02035821 |  | 0.8180088 | 0.824137 |  |  | 0.6324742 |  | 0.0708825 | 0.06846111 |
| P2 H24 | 0.6529903 | 0.8180088 |  | 0.01526876 |  |  | 0.1380058 | 0.0708825 |  | 0.6322246 |
| P3 H24 | 0.1054832 | 0.824137 | 0.01526876 |  |  |  | -0.4695366 | 0.06846111 | 0.6322246 |  |
|  |  |  |  |  |  |  |  |  |  |  |
| **Bounding box** | P2 H12 | P3 H12 | P2 H24 | P3 H24 |  | Coeff | P2 H12 | P3 H12 | P2 H24 | P3 H24 |
| P2 H12 |  | 0.1868972 | 0.2738391 | 0.1864046 |  |  |  | 0.3906606 | 0.3280502 | -0.3910621 |
| P3 H12 | 0.1868972 |  | 0.9037803 | 0.1809333 |  |  | 0.3906606 |  | 0.03727256 | 0.3955677 |
| P2 H24 | 0.2738391 | 0.9037803 |  | 0.2665041 |  |  | 0.3280502 | 0.03727256 |  | 0.3188586 |
| P3 H24 | 0.1864046 | 0.1809333 | 0.2665041 |  |  |  | -0.3910621 | 0.3955677 | 0.3188586 |  |
|  |  |  |  |  |  |  |  |  |  |  |
| **Compactness** | P2 H12 | P3 H12 | P2 H24 | P3 H24 |  | Coeff | P2 H12 | P3 H12 | P2 H24 | P3 H24 |
| P2 H12 |  | 0.09300467 | 0.3540645 | 0.682179 |  |  |  | 0.4849704 | 0.2800518 | 0.1257947 |
| P3 H12 | 0.09300467 |  | 0.7124465 | 0.2135797 |  |  | 0.4849704 |  | 0.1133106 | 0.3698329 |
| P2 H24 | 0.3540645 | 0.7124465 |  | 0.02838536 |  |  | 0.2800518 | 0.1133106 |  | 0.5838021 |
| P3 H24 | 0.682179 | 0.2135797 | 0.02838536 |  |  |  | 0.1257947 | 0.3698329 | 0.5838021 |  |
|  |  |  |  |  |  |  |  |  |  |  |
| **Eccenticity** | P2 H12 | P3 H12 | P2 H24 | P3 H24 |  | Coeff | P2 H12 | P3 H12 | P2 H24 | P3 H24 |
| P2 H12 |  | 0.1464986 | 0.3876611 | 0.9248829 |  |  |  | 0.4261505 | 0.2617497 | -0.0290744 |
| P3 H12 | 0.1464986 |  | 0.2524553 | 0.9185936 |  |  | 0.4261505 |  | -0.3421794 | 0.03151564 |
| P2 H24 | 0.3876611 | 0.2524553 |  | 0.144589 |  |  | 0.2617497 | -0.3421794 |  | 0.4107503 |
| P3 H24 | 0.9248829 | 0.9185936 | 0.144589 |  |  |  | -0.0290744 | 0.03151564 | 0.4107503 |  |
|  |  |  |  |  |  |  |  |  |  |  |
| **Equivalent**  **diameter** | P2 H12 | P3 H12 | P2 H24 | P3 H24 |  | Coeff | P2 H12 | P3 H12 | P2 H24 | P3 H24 |
| P2 H12 |  | 0.06742067 | 0.9737133 | 0.03161799 |  |  |  | 0.5217696 | 0.01016304 | -0.5959032 |
| P3 H12 | 0.06742067 |  | 0.9033396 | 0.8031871 |  |  | 0.5217696 |  | -0.037444 | -0.0767547 |
| P2 H24 | 0.9737133 | 0.9033396 |  | 0.01787467 |  |  | 0.01016304 | -0.037444 |  | 0.6206049 |
| P3 H24 | 0.03161799 | 0.8031871 | 0.01787467 |  |  |  | -0.5959032 | -0.0767547 | 0.6206049 |  |
|  |  |  |  |  |  |  |  |  |  |  |
| **Extent** | P2 H12 | P3 H12 | P2 H24 | P3 H24 |  | Coeff | P2 H12 | P3 H12 | P2 H24 | P3 H24 |
| P2 H12 |  | 0.2342293 | 0.1660441 | 0.942706 |  |  |  | 0.3547961 | 0.4082858 | -0.0221641 |
| P3 H12 | 0.2342293 |  | 0.6332618 | 0.153157 |  |  | 0.3547961 |  | 0.1463665 | 0.4198999 |
| P2 H24 | 0.1660441 | 0.6332618 |  | 0.04510533 |  |  | 0.4082858 | 0.1463665 |  | 0.5423473 |
| P3 H24 | 0.942706 | 0.153157 | 0.04510533 |  |  |  | -0.0221641 | 0.4198999 | 0.5423473 |  |
|  |  |  |  |  |  |  |  |  |  |  |
| **Form factor** | P2 H12 | P3 H12 | P2 H24 | P3 H24 |  | Coeff | P2 H12 | P3 H12 | P2 H24 | P3 H24 |
| P2 H12 |  | 0.4288318 | 0.2916751 | 0.9786019 |  |  |  | 0.2404139 | 0.316752 | -0.0082725 |
| P3 H12 | 0.4288318 |  | 0.7531887 | 0.2765937 |  |  | 0.2404139 |  | 0.09675346 | 0.326278 |
| P2 H24 | 0.2916751 | 0.7531887 |  | 0.01024586 |  |  | 0.316752 | 0.09675346 |  | 0.6597791 |
| P3 H24 | 0.9786019 | 0.2765937 | 0.01024586 |  |  |  | -0.0082725 | 0.326278 | 0.6597791 |  |
|  |  |  |  |  |  |  |  |  |  |  |
| **Major axis**  **length** | P2 H12 | P3 H12 | P2 H24 | P3 H24 |  | Coeff | P2 H12 | P3 H12 | P2 H24 | P3 H24 |
| P2 H12 |  | 0.2047971 | 0.5750999 | 0.1880444 |  |  |  | 0.3764994 | 0.1716 | -0.389728 |
| P3 H12 | 0.2047971 |  | 0.6231403 | 0.2186731 |  |  | 0.3764994 |  | -0.1506923 | 0.3660439 |
| P2 H24 | 0.5750999 | 0.6231403 |  | 0.2883851 |  |  | 0.1716 | -0.1506923 |  | 0.3053648 |
| P3 H24 | 0.1880444 | 0.2186731 | 0.2883851 |  |  |  | -0.389728 | 0.3660439 | 0.3053648 |  |
|  |  |  |  |  |  |  |  |  |  |  |
| **Maximum**  **feret diameter** | P2 H12 | P3 H12 | P2 H24 | P3 H24 |  | Coeff | P2 H12 | P3 H12 | P2 H24 | P3 H24 |
| P2 H12 |  | 0.2322098 | 0.6055411 | 0.1302224 |  |  |  | 0.3562301 | 0.1582768 | -0.4422578 |
| P3 H12 | 0.2322098 |  | 0.5641276 | 0.3026937 |  |  | 0.3562301 |  | -0.1764711 | 0.3099702 |
| P2 H24 | 0.6055411 | 0.5641276 |  | 0.2944613 |  |  | 0.1582768 | -0.1764711 |  | 0.3017252 |
| P3 H24 | 0.1302224 | 0.3026937 | 0.2944613 |  |  |  | -0.4422578 | 0.3099702 | 0.3017252 |  |
|  |  |  |  |  |  |  |  |  |  |  |
| **Maximum**  **radius** | P2 H12 | P3 H12 | P2 H24 | P3 H24 |  | Coeff | P2 H12 | P3 H12 | P2 H24 | P3 H24 |
| P2 H12 |  | 0.1020541 | 0.7376595 | 0.9416327 |  |  |  | 0.473648 | 0.1030337 | -0.0225799 |
| P3 H12 | 0.1020541 |  | 0.8620113 | 0.4975378 |  |  | 0.473648 |  | -0.0535713 | -0.2069446 |
| P2 H24 | 0.7376595 | 0.8620113 |  | 0.01181814 |  |  | 0.1030337 | -0.0535713 |  | 0.6502119 |
| P3 H24 | 0.9416327 | 0.4975378 | 0.01181814 |  |  |  | -0.0225799 | -0.2069446 | 0.6502119 |  |
|  |  |  |  |  |  |  |  |  |  |  |
| **Mean**  **radius** | P2 H12 | P3 H12 | P2 H24 | P3 H24 |  | Coeff | P2 H12 | P3 H12 | P2 H24 | P3 H24 |
| P2 H12 |  | 0.1039735 | 0.1977007 | 0.3713667 |  |  |  | 0.4713353 | 0.382017 | 0.2705163 |
| P3 H12 | 0.1039735 |  | 0.6295168 | 0.8497579 |  |  | 0.4713353 |  | 0.147964 | -0.0583754 |
| P2 H24 | 0.1977007 | 0.6295168 |  | 0.01304481 |  |  | 0.382017 | 0.147964 |  | 0.6434057 |
| P3 H24 | 0.3713667 | 0.8497579 | 0.01304481 |  |  |  | 0.2705163 | -0.0583754 | 0.6434057 |  |
|  |  |  |  |  |  |  |  |  |  |  |
| **Median**  **radius** | P2 H12 | P3 H12 | P2 H24 | P3 H24 |  | Coeff | P2 H12 | P3 H12 | P2 H24 | P3 H24 |
| P2 H12 |  | 0.04946245 | 0.09855188 | 0.1684981 |  |  |  | 0.5540203 | 0.4779453 | 0.4061407 |
| P3 H12 | 0.04946245 |  | 0.4968512 | 0.9131981 |  |  | 0.5540203 |  | 0.2072679 | 0.03361129 |
| P2 H24 | 0.09855188 | 0.4968512 |  | 0.01404135 |  |  | 0.4779453 | 0.2072679 |  | 0.6382287 |
| P3 H24 | 0.1684981 | 0.9131981 | 0.01404135 |  |  |  | 0.4061407 | 0.03361129 | 0.6382287 |  |
|  |  |  |  |  |  |  |  |  |  |  |
| **Minimum**  **feret diameter** | P2 H12 | P3 H12 | P2 H24 | P3 H24 |  | Coeff | P2 H12 | P3 H12 | P2 H24 | P3 H24 |
| P2 H12 |  | 0.00866839 | 0.9550908 | 0.8103086 |  |  |  | 0.6927602 | 0.01736811 | -0.0739304 |
| P3 H12 | 0.00866839 |  | 0.8296504 | 0.6677334 |  |  | 0.6927602 |  | 0.06628573 | 0.1318155 |
| P2 H24 | 0.9550908 | 0.8296504 |  | 0.00106588 |  |  | 0.01736811 | 0.06628573 |  | 0.7774227 |
| P3 H24 | 0.8103086 | 0.6677334 | 0.00106588 |  |  |  | -0.0739304 | 0.1318155 | 0.7774227 |  |
|  |  |  |  |  |  |  |  |  |  |  |
| **Minor axis**  **length** | P2 H12 | P3 H12 | P2 H24 | P3 H24 |  |  | P2 H12 | P3 H12 | P2 H24 | P3 H24 |
| P2 H12 |  | 0.00608038 | 0.9291953 | 0.8368522 |  |  |  | 0.7143618 | -0.0274013 | -0.0634484 |
| P3 H12 | 0.00608038 |  | 0.9855851 | 0.6913222 |  |  | 0.7143618 |  | 0.00557252 | 0.1220054 |
| P2 H24 | 0.9291953 | 0.9855851 |  | 0.00047682 |  |  | -0.0274013 | 0.00557252 |  | 0.807619 |
| P3 H24 | 0.8368522 | 0.6913222 | 0.00047682 |  |  |  | -0.0634484 | 0.1220054 | 0.807619 |  |
|  |  |  |  |  |  |  |  |  |  |  |
| **Perimeter** | P2 H12 | P3 H12 | P2 H24 | P3 H24 |  |  | P2 H12 | P3 H12 | P2 H24 | P3 H24 |
| P2 H12 |  | 0.08459282 | 0.5172908 | 0.4487017 |  |  |  | 0.4961943 | 0.1977282 | -0.2304853 |
| P3 H12 | 0.08459282 |  | 0.8443348 | 0.4060553 |  |  | 0.4961943 |  | 0.06050543 | 0.2520812 |
| P2 H24 | 0.5172908 | 0.8443348 |  | 0.02430866 |  |  | 0.1977282 | 0.06050543 |  | 0.5966129 |
| P3 H24 | 0.4487017 | 0.4060553 | 0.02430866 |  |  |  | -0.2304853 | 0.2520812 | 0.5966129 |  |
|  |  |  |  |  |  |  |  |  |  |  |
| **Solidity** | P2 H12 | P3 H12 | P2 H24 | P3 H24 |  |  | P2 H12 | P3 H12 | P2 H24 | P3 H24 |
| P2 H12 |  | 0.1886549 | 0.3030683 | 0.3195986 |  |  |  | 0.3892333 | 0.3097422 | 0.2998327 |
| P3 H12 | 0.1886549 |  | 0.6301583 | 0.06461181 |  |  | 0.3892333 |  | 0.1476901 | 0.5263751 |
| P2 H24 | 0.3030683 | 0.6301583 |  | 0.02645476 |  |  | 0.3097422 | 0.1476901 |  | 0.5896842 |
| P3 H24 | 0.3195986 | 0.06461181 | 0.02645476 |  |  |  | 0.2998327 | 0.5263751 | 0.5896842 |  |
|  |  |  |  |  |  |  |  |  |  |  |
| **1st closest**  **distance** | P2 H12 | P3 H12 | P2 H24 | P3 H24 |  |  | P2 H12 | P3 H12 | P2 H24 | P3 H24 |
| P2 H12 |  | 0.05128593 | 0.1773878 | 0.1546977 |  |  |  | -0.5503979 | 0.3985338 | -0.4184788 |
| P3 H12 | 0.05128593 |  | 0.9262299 | 0.05707227 |  |  | -0.5503979 |  | -0.0285517 | 0.5394796 |
| P2 H24 | 0.1773878 | 0.9262299 |  | 0.2566097 |  |  | 0.3985338 | -0.0285517 |  | -0.3251772 |
| P3 H24 | 0.1546977 | 0.05707227 | 0.2566097 |  |  |  | -0.4184788 | 0.5394796 | -0.3251772 |  |
|  |  |  |  |  |  |  |  |  |  |  |
| **2nd closest**  **distance** | P2 H12 | P3 H12 | P2 H24 | P3 H24 |  |  | P2 H12 | P3 H12 | P2 H24 | P3 H24 |
| P2 H12 |  | 0.1976855 | 0.2845283 | 0.9339038 |  |  |  | -0.382029 | 0.3212297 | -0.0255754 |
| P3 H12 | 0.1976855 |  | 0.7669567 | 0.3934143 |  |  | -0.382029 |  | -0.0912144 | 0.2587006 |
| P2 H24 | 0.2845283 | 0.7669567 |  | 0.382338 |  |  | 0.3212297 | -0.0912144 |  | -0.2532496 |
| P3 H24 | 0.9339038 | 0.3934143 | 0.382338 |  |  |  | -0.0255754 | 0.2587006 | -0.2532496 |  |
|  |  |  |  |  |  |  |  |  |  |  |

S4 Appendix: Comparison of the cell shape characteristics between defrosted cells (P2) and sub-cultured cells (P3) at 12 and 24 hours (H) of imaging. *: p<0.05


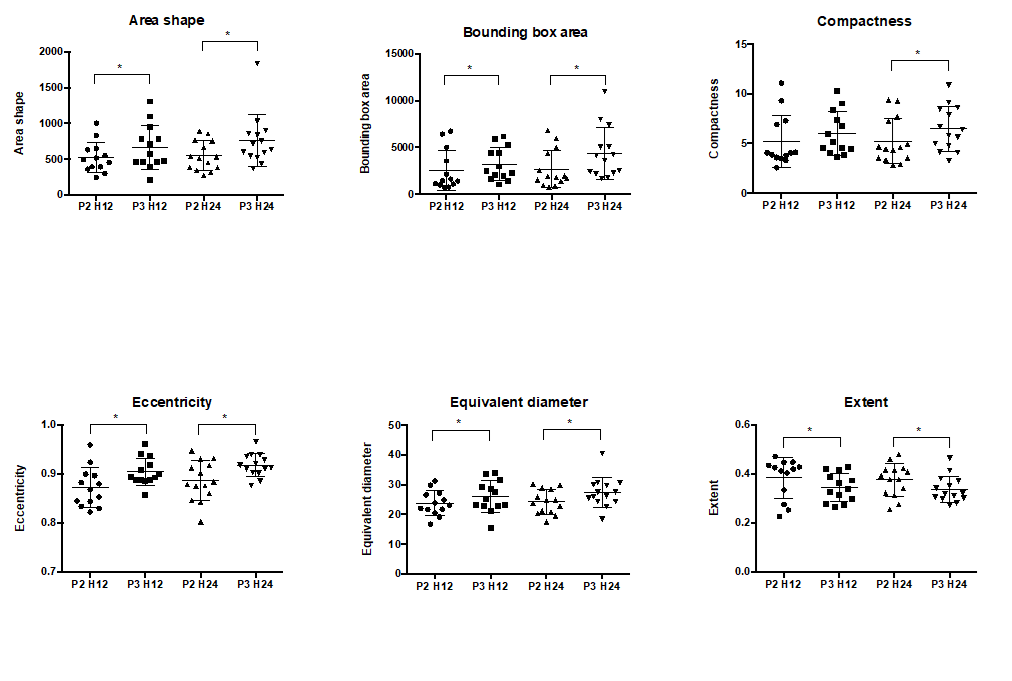


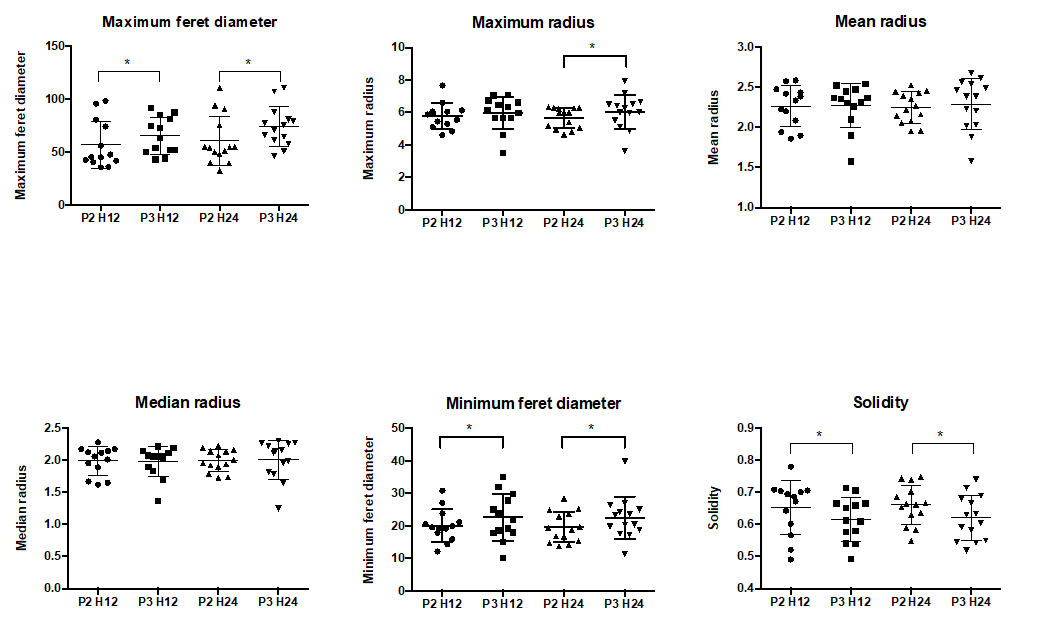


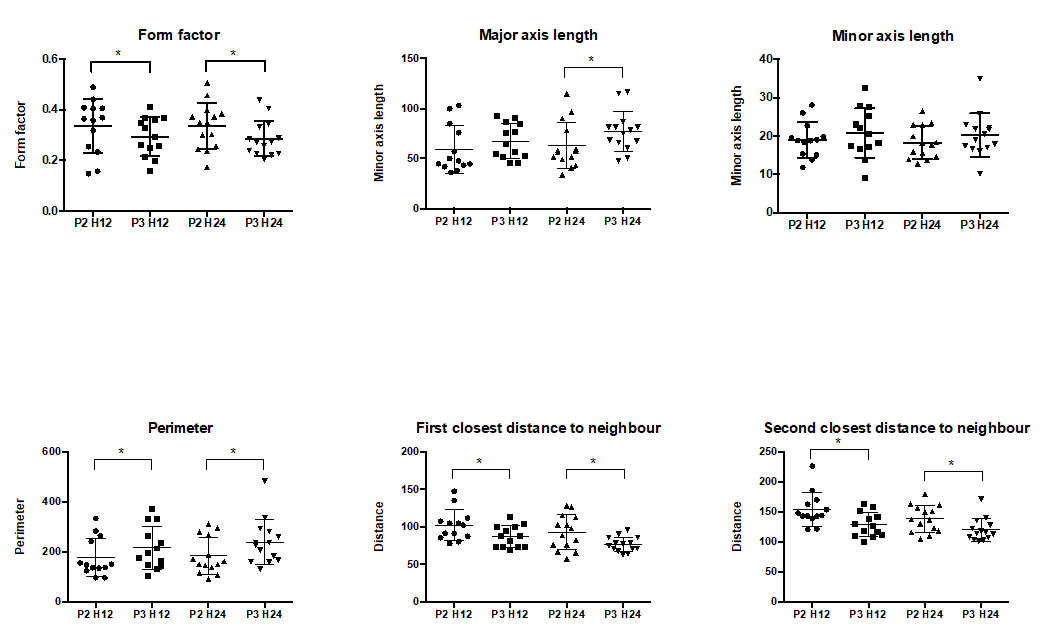

Supplement: sj-docx-1-tej-10.1177_20417314221139794 – Supplemental material for Cell shape characteristics of human skeletal muscle cells as a predictor of myogenic competency: A new paradigm towards precision cell therapy [file sj-docx-1-tej-10.1177_20417314221139794.docx]
